# Identification and Validation of Necroptosis-Related LncRNA Signature in Hepatocellular Carcinoma for Prognosis Estimation and Microenvironment Status

**DOI:** 10.3389/fgene.2022.898507

**Published:** 2022-06-08

**Authors:** Cong Chen, Yumeng Wu, Kang Chen, Zicong Xia, Xiaokan Liu, Chaojie Zhang, Hui Zhao, Aiguo Shen

**Affiliations:** ^1^ Department of Interventional Radiology, Affiliated Hospital of Nantong University, Nantong, China; ^2^ Cancer Research Center Nantong, Affiliated Tumor Hospital of Nantong University, Nantong, China

**Keywords:** hepatocellular carcinoma, prognostic signature, microenvironment, lncRNA, necroptosis, immune infiltrate

## Abstract

**Background:** Hepatocellular carcinoma (HCC) is among malignancies with the highest fatality toll globally and minimal therapeutic options. Necroptosis is a programmed form of necrosis or inflammatory cell death, which can affect prognosis and microenvironmental status of HCC. Therefore, we aimed to explore the prognostic value of necroptosis-related lncRNAs (NRLs) in HCC and the role of the tumor microenvironment (TME) in immunotherapy.

**Methods:** The RNA-sequencing data and clinical information were downloaded from The Cancer Genome Atlas (TCGA) and the International Cancer Genome Consortium (ICGC). NRLs were identified by Pearson correlation analysis. The signature was constructed using the LASSO–Cox regression analysis and evaluated using the receiver operating characteristic curve (ROC) and the area under the Kaplan–Meier curve. The nomogram was built based on clinical information and risk score. Gene set enrichment analysis (GSEA), immunoassay, half-maximum inhibitory concentration (IC_50_) analysis of the risk group, and the HCC subtype identification based on NRLs were also carried out. Finally, we detected the expression of lncRNAs in HCC tissues and cell lines *in vitro*.

**Results:** A total of 508 NRLs were screened out, and seven NRLs were constructed as a risk stratification system to classify patients into distinct low- and high-risk groups. Patients in the high-risk group had a significantly lower overall survival (OS) than those in the low-risk group. Using multivariate Cox regression analysis, we found that the risk score was an independent predictor of OS. Functional analysis showed that the immune status of different patients was different. The IC_50_ analysis of chemotherapy demonstrated that patients in the high-risk group were more sensitive to commonly prescribed drugs. qRT-PCR showed that three high-risk lncRNAs were upregulated in drug-resistant cells, and the expression in HCC tissues was higher than that in adjacent tissues.

**Conclusion:** The prediction signature developed in this study can be used to assess the prognosis and microenvironment of HCC patients, and serve as a new benchmark for HCC treatment selection.

## Introduction

According to the 2020 global cancer statistics, primary liver cancer ranks the sixth and the third in the worldwide incidence rate and mortality rate, respectively, and hepatocellular carcinoma (HCC) accounts for the overwhelming majority of liver cancer cases (Sung et al., 2021). Even though early diagnosis of HCC has developed rather rapidly in recent years, in most patients HCC is already in the intermediate or advanced stage at the time of diagnosis, having missed the best time for surgical resection. New treatments, such as transcatheter arterial chemoembolization (TACE), radiofrequency ablation, immunotherapy, and targeted therapy, can bring hope to patients with advanced liver cancer (Anwanwan et al., 2020). However, the overall survival (OS) of patients with HCC remains unsatisfactory, and changes in the immune microenvironment possibly play a pivotal role in immune escape and resistance in HCC. In recent years, immunotherapy—represented by immune checkpoint inhibitors, adoptive cell therapy (ACT), and tumor vaccines—has brought new hope to patients with advanced HCC (Mizukoshi and Kaneko, 2019). However, only a small fraction of patients can benefit from immunotherapy (Fu et al., 2019), which may be due to tumor heterogeneity and changes in immune-related factors in the tumor microenvironment (TME). Therefore, finding an indicator that can predict the effect of immunotherapy and the state of the microenvironment is crucial for improving the prognosis in patients with HCC.

Cell death is a complex process, that is, achieved through pathological and physiological ways (Tang et al., 2019). One form of cell death is necroptosis, which can be acquired in a programmed way during the development of certain organisms. Unlike typical apoptosis and necrosis, necroptosis induces cell death when the apoptotic mechanism fails. Necroptosis is closely related to the immune microenvironment and can induce cell rupture, activate inflammatory response while releasing cellular contents, and promote infiltration of many inflammatory cells (Green, 2019). Receptor-interacting serine/threonine-protein kinase 3 (RIPK3) can act as a critical regulator of necroptosis to affect the function of immune cells by regulating the activation of natural killer T (NKT) cells and dendritic cells (DCs) (Degterev et al., 2008). The pan-caspase inhibitor Z-VAD (OH)-FMK (zVAD) has been reported to induce necroptosis in melanoma *via* reducing tumor infiltration by regulatory T cells (Tregs) while increasing DC and CD8^+^ T cells to reduce tumor growth (Werthmöller et al., 2015). Necroptosis also plays a vital role in HCC since heparinase can induce necrotic proliferation of microvascular endothelial cells and promote liver cancer metastasis (Chen et al., 2021). Hence, necroptosis may be a potential target for HCC therapy.

Long noncoding RNAs (lncRNAs) are a class of noncoding RNAs. lncRNAs are closely related to HCC. Specifically, downregulation of lncRNA growth arrest-specific 5 (GAS5) in HCC promotes proliferation and drug resistance through the decrease of phosphatase and tensin homolog (PTEN) expression (Wang et al., 2020). lncRNA small nucleolar RNA host gene 3 (SNHG3) induces epithelial–mesenchymal transition (EMT) and sorafenib resistance by regulating the miR-128/cluster of differentiation 151 (CD151) pathway in HCC (Zhang et al., 2019), having the potential to affect necroptosis through different pathways such as H19-derived miR-675 targeting FAS-associated death domain protein (FADD) (Harari-Steinfeld et al., 2021). In addition, lncRNAs can protect tumor cells from necroptosis by suppressing the expression of some related proteins (Tao et al., 2019). There is also a close correlation between lncRNAs and TME. Long intergenic non-protein coding RNA 665 (LINC00665) affects the level of macrophage and DC infiltration, suppresses Tregs, and prevents T cell failure by targeting lncRNA five prime to Xist (FTX) as competing endogenous RNA (ceRNA) (Zhang et al., 2020a). lncRNA T cell leukemia/lymphoma 6 (TCL6) positively correlates with tumor-infiltrating lymphocyte (TIL) infiltration and immune checkpoint molecules such as cytotoxic T-cell lymphocyte-associated protein 4 (CTLA-4), programmed death receptor 1 (PD-1), and its ligand (PD-L1) (Zhang et al., 2020b). Exploring the lncRNA signatures associated with necroptosis and their role in HCC treatment requires special attention.

In this study, we first downloaded lncRNA expression profiles and clinical information from The Cancer Genome Atlas (TCGA) and the International Cancer Genome Consortium (ICGC); then, we constructed a necroptosis-related lncRNA prognostic signature, which allowed us to analyze TME, immune cell infiltration, immune checkpoints, human leukocyte antigens (HLA), functional enrichment, and drug sensitivity in different risk groups. Finally, we validated the lncRNAs in the signature using tissues and cell lines. This study may provide a new reference for selecting HCC treatment methods and predicting prognosis.

## Materials and Methods

### Datasets and Preprocessing

The RNA-sequencing data (TPM format) used for HCC samples were downloaded from TCGA (https://portal.gdc.cancer.gov/). After excluding the patients from repeated sequencing, those lacking complete follow-up information, and those with 0 survival days, a total of 50 normal samples and 365 tumor samples were included. Next, bioinformatics analysis, survival analysis, and model building were performed on these samples. Data from 231 HCC patients were additionally downloaded from ICGC (https://dcc.icgc.org/projects/LIRI-JP) for external validation using the same exclusion criteria. The “SVA” R package was used to perform background correction, normalization, and expression estimates for internal and external validation on the genes associated with the modeling (Supplementary Figure S1). R software (version 4.0.5) was used to conduct all of the analyses.

### Construction and Validation of Prognostic Signature

Necroptosis-related genes (NRGs) were extracted from previous studies (Supplementary File S1) (Zhao et al., 2021). Differential expression of NRGs in normal and HCC samples was analyzed using the limma R package, with *p* < 0.05 and | log_2_FC| > 0.5 as thresholds. Having performed the Pearson correlation analysis on all lncRNAs and having identified differentially expressed NRGs (*p* < 0.001, correlation coefficient >0.4), we finally screened necroptosis-related lncRNAs (NRLs) for subsequent bioinformatics analysis.

Univariate Cox proportional-hazard regression analysis filtered lncRNAs linked to survival (*p* < 0.05) in the batch-adjusted cohort. A risk model was then built using Least Absolute Shrinkage and Selection Operator (LASSO) regression with 10-fold cross-validation and run for 1,000 cycles with 1,000 random stimulations to avoid overfitting effects (Tibshirani, 1997; Simon et al., 2011). After integrating the gene expression values weighted by the LASSO–Cox coefficient, the following formula for the risk score was established:

risk score=∑[Exp(IncRNA)×coef(IncRNA)] (1)

where Exp (lncRNA) is the expression of survival-related lncRNAs, and coef (lncRNA) is the associated regression coefficient. Patients in the TCGA and ICGC cohorts were divided into high- and low-risk groups based on the median risk score. Kaplan–Meier (K-M) curves were plotted to find differences in OS between the risk groups, and log-rank tests were performed on the results. Likewise, receiver operating characteristic (ROC) curves were plotted using the survival ROC R package. The area under the curve (AUC) was calculated to assess the model’s accuracy.

Finally, we analyzed the clinicopathological information in the dataset through univariate and multivariate Cox regression analysis. We used a nomogram that included tumor-node-metastasis (TNM) staging and risk score to predict the survival of HCC patients at 1, 3, and 5 years. The nomogram’s accuracy was measured using ROC curves. The *p*-values in analyzing the differentially expressed genes were adjusted.

### Immunology and Cluster Analysis

We utilized different algorithms such as TIMER, CIBERSORT, CIBERSORT-abs, QUANTISEQ, MCP-counter, XCELL, and EPIC to estimate the abundance and correlation of immune cells in different risk groups. In addition, the single-sample gene set enrichment analysis (ssGSEA) algorithm was selected to evaluate immune cells and immune-related functions (Rooney et al., 2015). The enrichment fraction of 29 immunological characteristics per sample in TME was calculated using the R package GSVA (version 1.34.0). The estimation of stromal and immune cells in malignant tumor tissues using the expression data (ESTIMATE) algorithm was employed to calculate the immune score, stromal score, and tumor purity to reflect the state of the immune microenvironment (Yoshihara et al., 2013). Regarding the TCGA cohort, we used a nonnegative matrix factorization (NMF) clustering algorithm to cluster the HCC samples from the NRLs. The ICGC cohort was verified using the same candidate genes. The K value refers to the value selected when the size of the correlation coefficient starts to decrease with the optimal number of clusters. The class mapping analysis evaluated the similarity of subtype classification among different datasets. Simultaneously, the dimensionality reduction analysis was performed on the expression data of the candidate genes, and the principal component analysis (PCA) method was adopted to verify the subtype distribution. In addition, the nearest template prediction (NTP) algorithm was applied to predict the different risk groups of genetic signatures in both cohorts. The prediction results were compared with the classification results of the NMF algorithm.

### Functional Enrichment Analysis

Gene pathways were annotated with the Kyoto Encyclopedia of Genes and Genomes (KEGG) and Gene Ontology (GO) using the “clusterProfiler” software R package. *p*-value < 0.05 and q-value < 0.05 indicated significantly enriched pathways. The Gene Set Enrichment Analysis (GSEA) algorithm is an enrichment method based on expression profiles, and calculates the estimated proportion of a particular pathway or feature in different clusters. We used the gene set (Kegg.v7.4.symbols.gmt) for GSEA analysis (http://www.gsea-msigdb.org/gsea/index.jsp), where *p* < 0.05 and false-discovery rate (FDR) < 0.05 were considered statistically significant. One thousand permutations of gene sets were done for each analysis to provide a normalized enrichment score (NES). The Benjamini–Hochberg (BH) multiple testing correction was used to adjust the *p*-values.

### Drug Sensitivity Analysis

Half-maximum inhibitory concentration (IC_50_) values of chemotherapy drugs were obtained from the Genomics of Cancer Drug Sensitivity (GDSC) database (https://www.cancerrxgene.org/) (Geeleher et al., 2014) and calculated using the “PRrophytic” R package in R software. The difference in the IC_50_ between the different risk groups was analyzed by the Wilcoxon signed-rank test. The results are shown as box plots.

### Cell Lines and Culture Conditions

All cell lines were purchased from the National Certified Cell Culture Collection Center (Shanghai, China). Huh7 and HepG2 cells were cultured in DMEM medium (Gibco) supplemented with 10% fetal bovine serum and 1% penicillin–streptomycin. Hep3B cells were cultured in MEM medium (HyClone), supplemented with 10% fetal bovine serum, 1% penicillin–streptomycin, and 1% non-essential amino acids (Gibco, #11140050). SNU-387 and L-02 cells were cultured in RPMI medium (Gibco) supplemented with 10% fetal bovine serum and 1% penicillin–streptomycin. Cell culture took place in a cell incubator at 37°C under 5% carbon dioxide and 10% humidity conditions. None of the cell lines used in this study were tested for mycoplasma contamination.

### Cell Viability and Drug Sensitivity

Cells were seeded in 96-well plates at a density of 5,000 cells/well and placed in a 37°C, 5% CO_2_ incubator for 24 h. We added doxorubicin (MCE, #HY-15142A), cisplatin (MCE, #HY-17394), and sorafenib (MCE, #HY-10201) to the experimental group according to the concentration gradient. After 48 h, the plates were removed from the incubator and placed in a dark environment to add 10 μl of CCK8 reagent (Vazyme, #A311-02) to each well. Then, the plates were returned to the incubator for 1–2 h. The optical density (OD) value was measured with a microplate reader (Thermo, Multiskan FC), and GraphPad (Version 9.3.1.471) was used to calculate the IC_50_ value after the exportation of data.

### Quantitative Real-Time PCR

We obtained 12 pairs of HCC tissues and paracancerous tissues from the Department of Pathology, the Affiliated Cancer Hospital of Nantong University. Total RNAs from the tissue samples and cell lines were extracted using an RNA isolation kit (Vazyme, #RC112-01), and were then used to synthesize complementary DNA (cDNA) with the help of the cDNA Synthesis Kit (Vazyme, #R233-01) in line with the manufacturer’s instructions. Quantitative real-time PCR (qRT-PCR) was conducted on the SteponePlus (Applied Biosystems) using SYBR qPCR Master Mix (Vazyme, #Q511-02) and 10 μM primers. Relative expression values were normalized to the control gene (GADPH). The primer pairs used in this study are shown in Table 1.

**TABLE 1:** PCR primer sequences.

Gene	Primers
HCG27	F: CAG​CCC​TGG​GTG​GAG​ATT​TAA​GAT​G
	R: AGG​TGG​GTG​GGA​AGA​GGT​GTT​AC
C2orf27A	F: CAT​GCG​GTC​CTC​CAG​GTT​CAA​C
	R: CTC​TGC​CAA​CCA​ACT​GCC​CAT​C
BACE1-AS	F: TGG​CTG​TTG​CTG​AAG​AAT​GTG​ACT​C
	R: CAA​CCT​TCG​TTT​GCC​CAA​GAA​AGT​G
SNHG4	F: AAC​TCC​TGA​CCT​TGC​GAT​TTG​CC
	R: GAG​GTT​GTA​GTG​AGC​CGA​GAT​TGC
MIR210HG	F: AAT​AAC​CAA​GCC​GAG​TTG​CCT​CTG
	R: TCT​GGA​GCA​CAC​AAA​GGG​AAC​AAG
SNHG3	F: CAG​CCG​TTA​AGC​CAT​TTG​GAA​CTT​G
	R: CAA​CCC​TGA​CCT​CAA​CAC​CTT​GG
HCG11	F: CTG​AGG​CAG​GAG​AAT​CAC​TTG​AAC​C
	R: TGA​GAT​GGA​GTC​TTG​CTG​TGT​TGC

## Results

**FIGURE 1:**
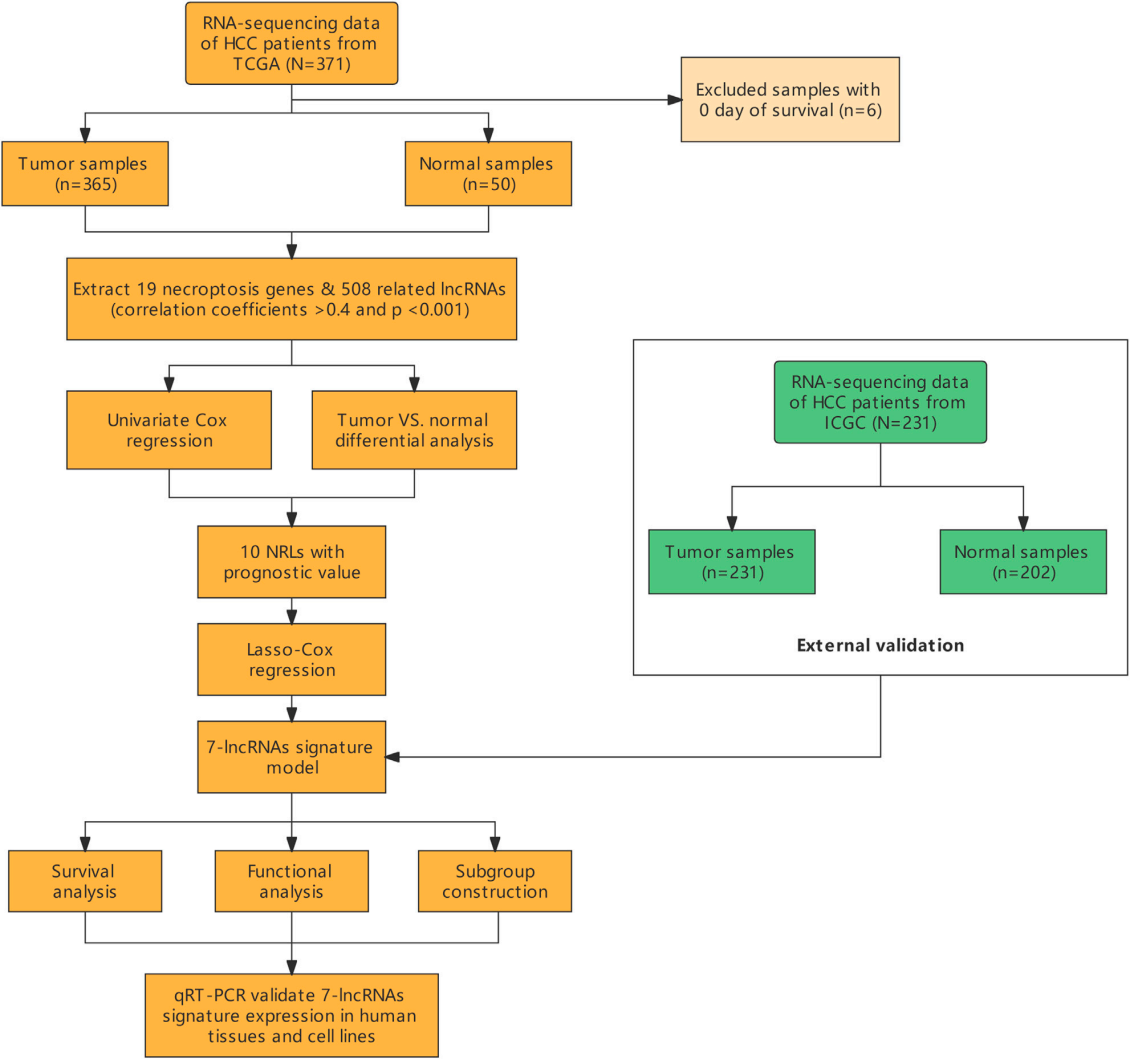
Flow chart of the study.

A total of 365 HCC patients from the TCGA cohort and 231 HCC patients from the ICGC (LIRI-JP) cohort were finally enrolled. The overview of this study is presented as a flowchart in Figure 1.

### The Landscape of Necroptosis-Related Genes in The Cancer Genome Atlas Cohort

**FIGURE 2:**
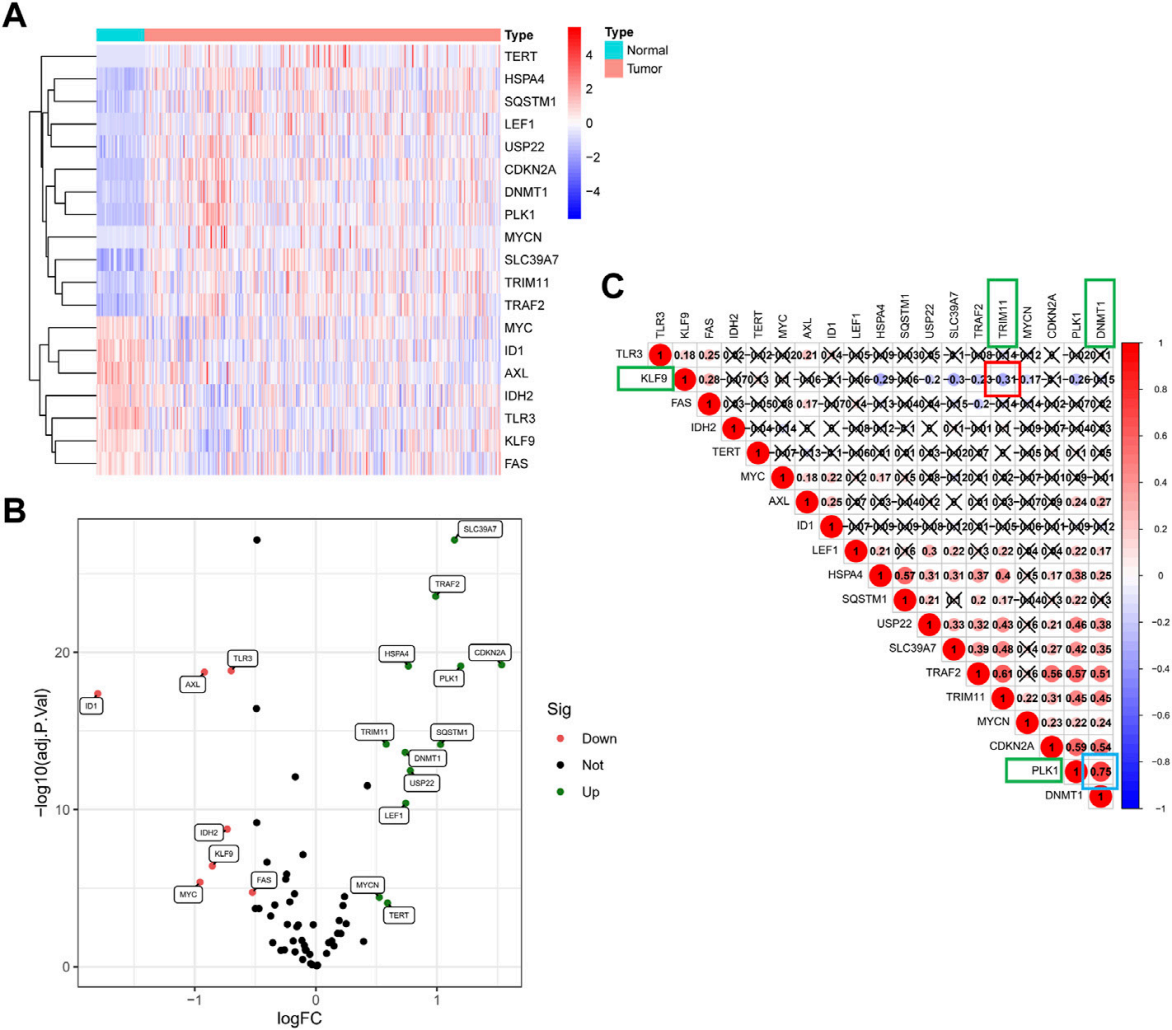
The landscape of NRGs in the TCGA cohort. **(A)** Heat map of the differentially expressed mRNAs in tumor tissues and adjacent normal tissues. **(B)**The volcano plot of 19 differentially expressed genes. **(C)** Correlation analysis of 19 NRGS revealed that DNMT1 has the strongest positive correlation with PLK1 (r = 0.75), and TRIM11 has the strongest negative correlation with KLF9 (r = −0.31).

In total, 19 of 67 NRGs showed significant differences in expression (Figure 2A); specifically, 12 genes were upregulated, and seven genes were downregulated (Figure 2B) (Supplementary File S2). As shown in Figure 2C, the correlation analysis of the 19 NRGs showed that DNA methyltransferase 1 (DNMT1) had the strongest positive correlation with polo-like kinase1 (PLK1) (r = 0.75) and that tripartite motif-containing protein 11 (TRIM11) had the strongest negative correlation with kruppel-like factor 9 (KLF9) (r = −0.31).

### Identification and Validation of Necroptosis-Related Long Noncoding RNAs

**FIGURE 3:**
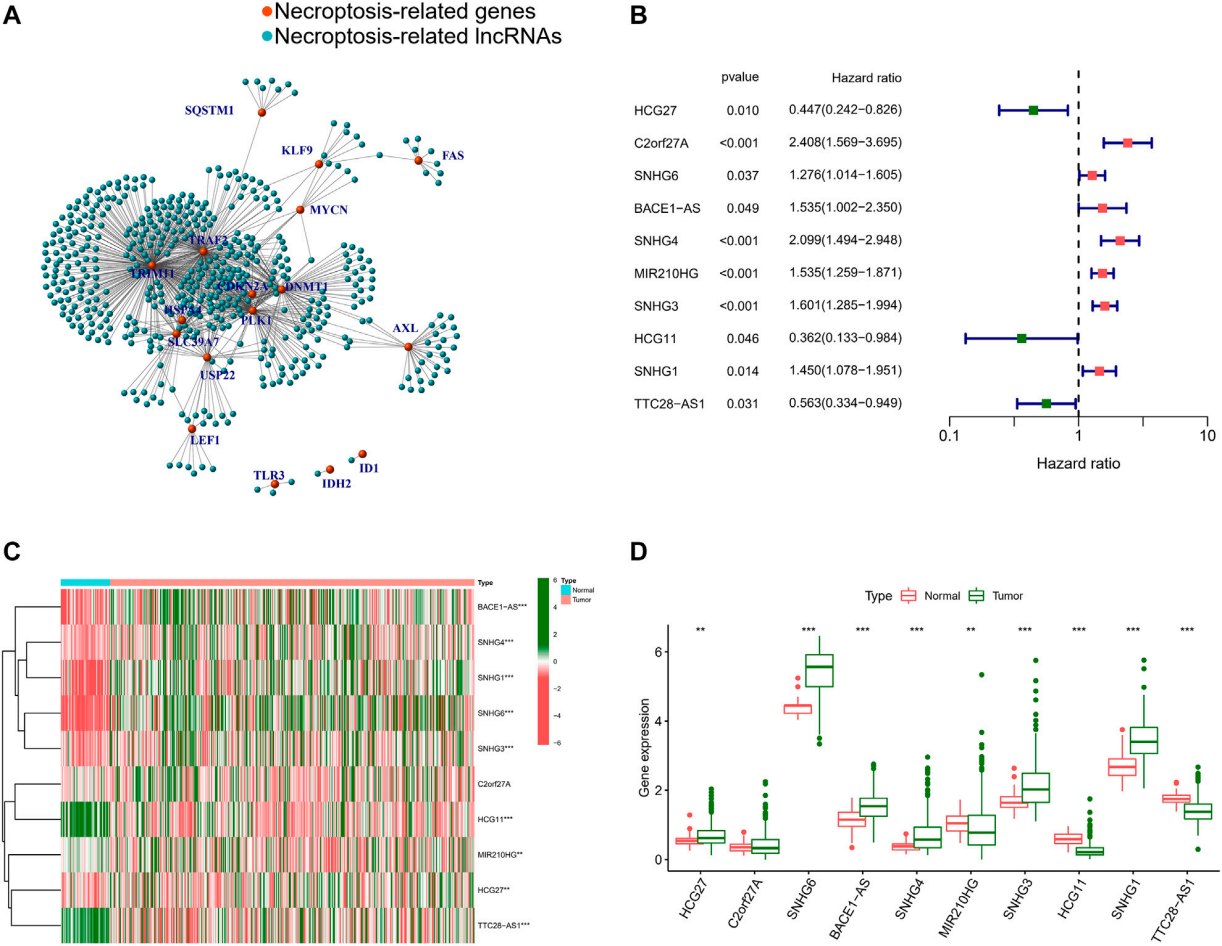
Identification of NRLs. **(A)** The network between NRGs and NRLs (correlation coefficients >0.4 and *p* < 0.001). **(B)** The prognostic NRLs were extracted by univariate Cox regression analysis. **(C)** Heatmap of prognostic NRLs in tumour tissues and adjacent normal tissues. **(D)** The expression of prognostic NRLs in tumour tissues and adjacent normal tissues. (*, *p* < 0.05; **, *p* < 0.01; ***, *p* < 0.001).

Having analyzed the correlation between the 19 NRGs and all annotated lncRNAs, we obtained 365 tumor samples and 50 normal samples from the TCGA cohort. Finally, we identified 508 NRLs (correlation coefficient >0.4 and *p* < 0.001), as presented in Figure 3A, showing the network diagram between NRGs and lncRNAs. Univariate Cox regression analysis was performed in the batch-adjusted TCGA-HCC cohort to determine the NRLs and their association with survival. Finally, 10 NRLs were screened for subsequent analysis (all *p* < 0.05) (Figure 3B). The Wilcoxon test showed that HLA complex group 27 (HCG27), small nucleolar RNA host gene 6 (SNHG6), antisense transcript of BACE1 (BACE1-AS), small nucleolar RNA host gene 4 (SNHG4), small nucleolar RNA host gene 3 (SNHG3), and small nucleolar RNA host gene 1 (SNHG1) were highly expressed in tumors, while MIR210 host gene (MIR210HG), HLA complex group 11 (HCG11), and TTC28 antisense RNA 1 (TTC28-AS1) were highly expressed in normal samples (Figures 3C,D).

### Construction of the Risk Signature

**FIGURE 4:**
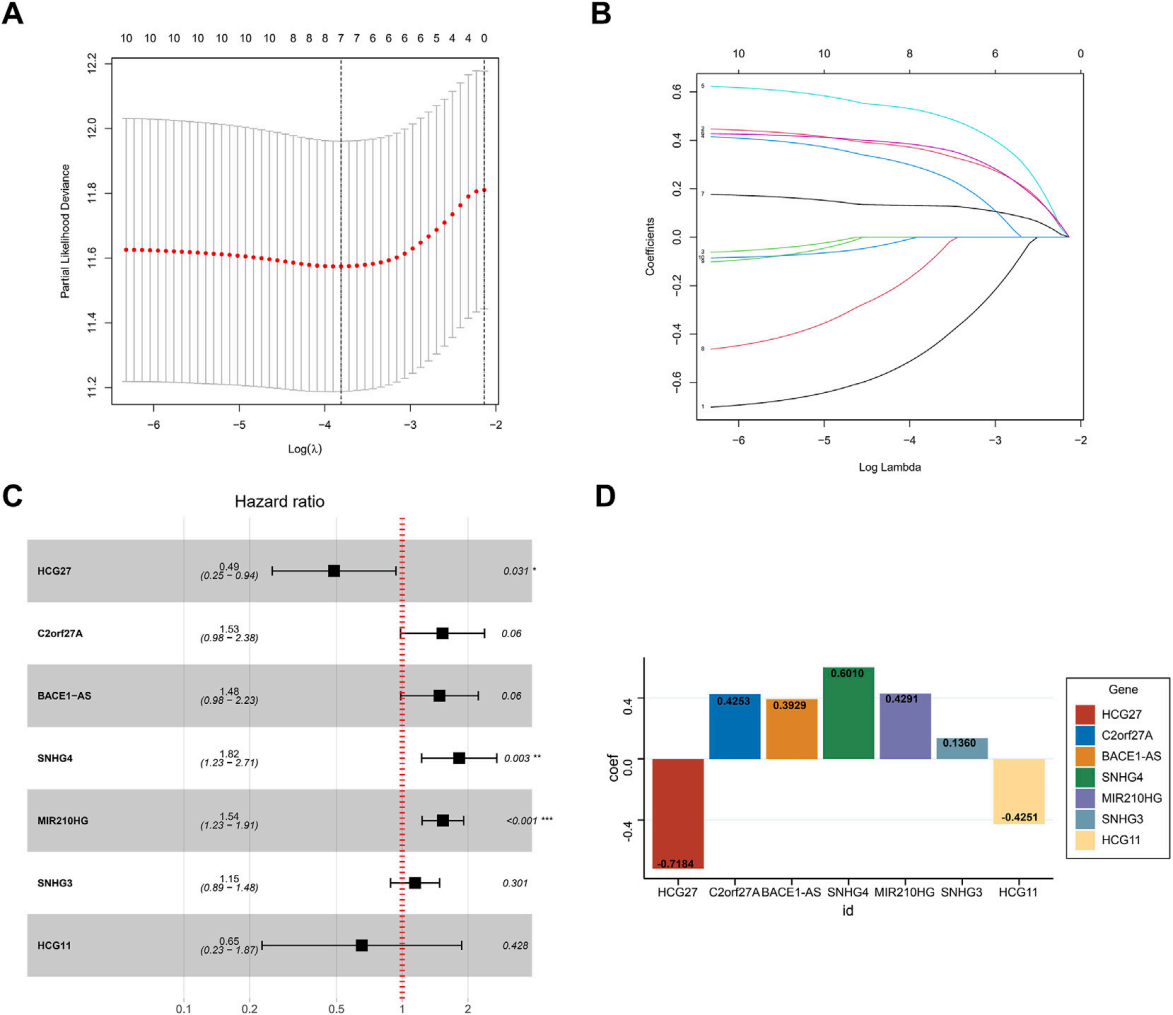
Construction of the prognostic signature. **(A)** The LASSO coefficient profiles of seven NRLs. **(B)** The ten-fold cross-validation for variable selection in the LASSO model. **(C)** Multivariate Cox analysis of the seven NRLs (ENTER method). **(D)** The regression coefficient of the seven NRLs in the signature.

Based on the optimal value of λ, we performed LASSO regression analysis on these 10 prognosis-related NRLs and screened seven NRLs (Figures 4A,B) to avoid overfitting of the prognostic signature. Then, we used multiple Cox regression analysis (ENTER method) to construct a risk stratification system, and showed that HCG27 and HCG11 were moderate-risk genes (Figure 4C). Finally, by combining the expression levels and regression coefficients of the seven NRLs (Figure 4D), we were able to derive the formula for the risk score of HCC patients: risk score = (−0.7184 × HCG27) + (0.4253 × C2orf27A) + (0.3929 × BACE1-AS) + (0.6010 × SNHG4) + (0.4291 × MIR210HG) + (0.1360 × SNHG3) + (−0.4251 × HCG11).

### Validation of the Risk Signature

**FIGURE 5:**
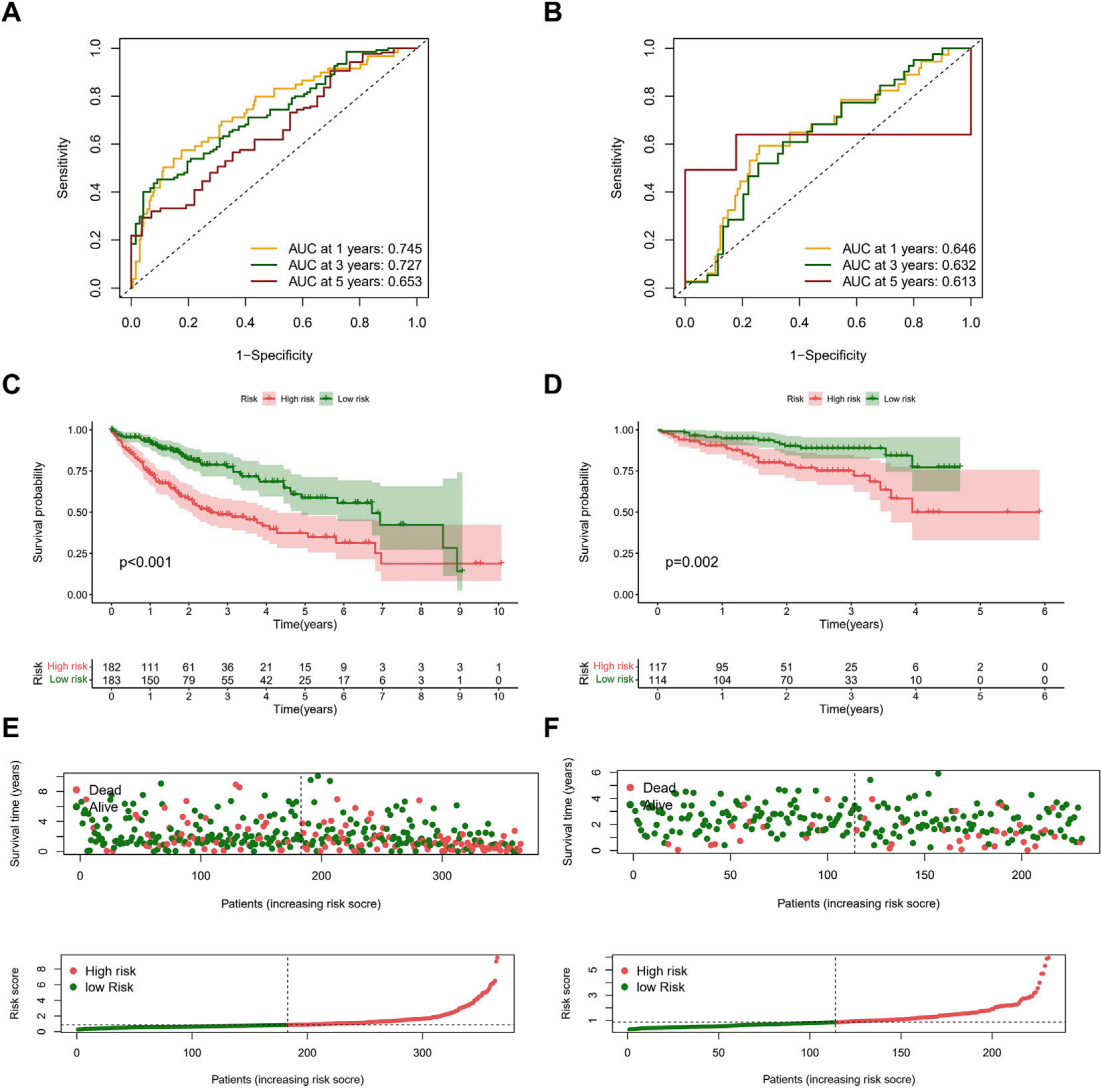
Evaluation of prognostic signature in the TCGA and ICGC cohorts. **(A)** ROC curves of the NRLs signature in the TCGA cohort. The AUCs of 1, 3, and 5 years OS were 0.745, 0.727, and 0.653. **(B)** ROC curves of the NRLs signature in the ICGC cohort. The AUCs of 1, 3, and 5 years OS were 0.646, 0.632, and 0.613. **(C,D)** Kaplan–Meier survival curves of OS (survival probability) of patients between different risk groups in the TCGA **(C)** and ICGC **(D)** cohorts. **(E,F)** Scatter plot (up) and curve plot (down) of risk score in the TCGA **(E)** and ICGC **(F)** cohorts.

We calculated the risk scores for patients in the TCGA cohort based on the risk score formula. We selected 231 tumor samples and 202 normal samples from the ICGC cohort as a validation set to test the stability of the signature. We then evaluated the predictive performance for OS using time-dependent ROC curves; the AUC for the TCGA cohort was 0.745, 0.727, and 0.653 at 1, 3, and 5 years, respectively (Figure 5A). The AUC of the ICGC cohort was 0.646, 0.632, and 0.613 in the same periods (Figure 5B). Kaplan–Meier curves showed that the OS of patients in the high-risk group was significantly lower than that in the low-risk group in both cohorts (all *p* < 0.01) (Figures 5C,D). In addition, we compared the risk score distribution and survival status of the high-risk group and the low-risk group using the risk score formula (Figures 5E,F).

**FIGURE 6:**
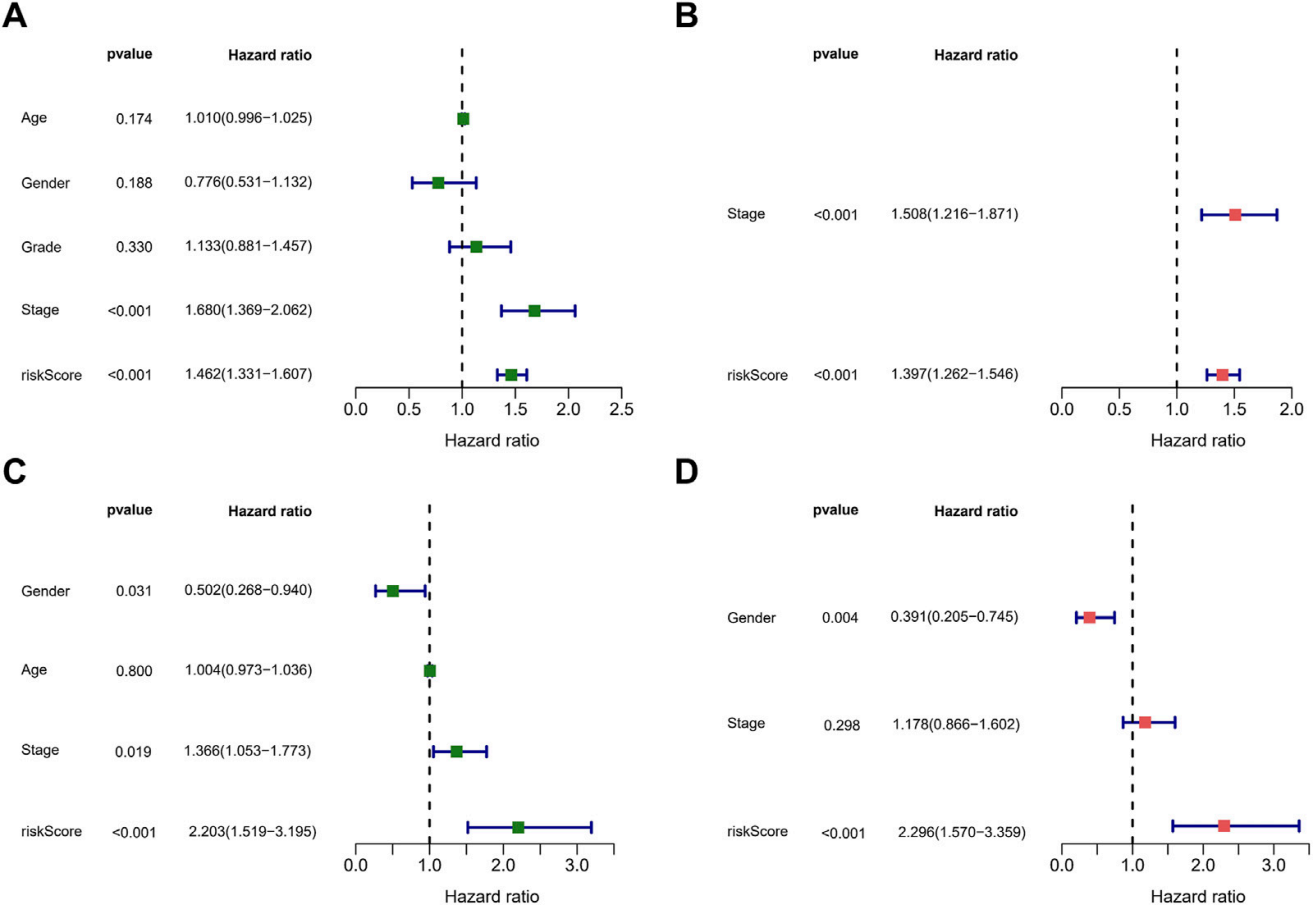
Assessment of the prognostic signature. **(A,C)** Univariate analysis of risk score and clinical characters in the TCGA **(A)** and ICGC **(C)** cohorts. **(B,D)** Multivariate analysis of risk score and clinical characters in the TCGA **(B)** and ICGC **(D)** cohorts.

Afterward, we performed univariate and multivariate Cox regression analysis on clinical characteristics and risk score to determine whether the risk score could serve as an independent prognostic factor for OS in HCC patients. Based on univariate Cox regression analysis, there was a significant association between the risk score and OS (TCGA cohort: HR = 1.462, 95% CI = 1.331–1.607; ICGC cohort: HR = 2.203, 95% CI = 1.519–3.195) (Figures 6A,C). After adjusting for other confounding factors, the risk score proved to be an independent predictor of OS in the multivariate Cox regression analysis (TCGA cohort: HR = 1.397, 95% CI = 1.262–1.546; ICGC cohort: HR = 2.296, 95% CI = 1.570–3.359) (Figures 6B,D). The hazard ratio (HR) and 95% confidence interval (CI) of the tumor stage in the multivariate Cox regression analysis of the TCGA cohort were 1.508 and 1.216–1.871 (*p* < 0.001), respectively. We believe that the TNM stage can also be considered an independent predictor.

### Construction of a Nomogram

**FIGURE 7:**
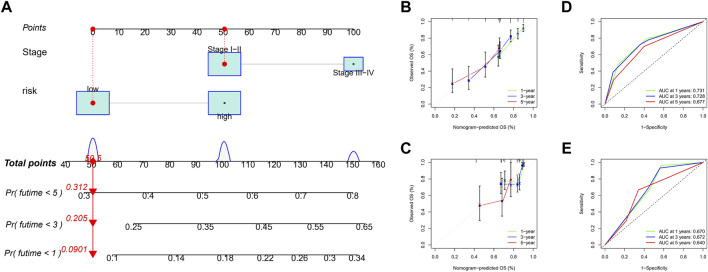
Construction and validation of nomogram. **(A)** The nomogram integrated the risk score and TNM stage to predict the survival rate of the 1, 3, and 5 years. **(B,C)** The 1, 3, and 5 years OS calibration curves for the TCGA **(B)** and ICGC **(C)** cohorts. **(D,E)** The 1, 3, and 5 years ROC curves of the TCGA **(D)** and ICGC **(E)** cohorts.

Considering the complexity of the risk signature, we visualized the risk signature by constructing a nomogram based on the risk score and TNM stage (Figure 7A). We used calibration curves for the TCGA and ICGC cohorts to verify the consistency of the nomogram in predicting the patients’ 1-, 3-, and 5-year OS. The prediction curves for both cohorts were close to the standard curve (Figures 7B,C), meaning that the nomogram can predict the patients’ OS quite well. Finally, we used the ROC curve to evaluate the sensitivity and specificity of the constructed risk signature for prognosis. The results showed that in the TCGA cohort, the areas under the ROC curve were 0.731, 0.728, and 0.677 at 1, 3, and 5 years (Figure 7D). In the ICGC cohort, the areas under the ROC curve were 0.670, 0.672, and 0.640 at the same time points (Figure 7E). In summary, the risk model showed an excellent predictive potential.

### Immune Phenotype Landscape in the Tumor Microenvironment of Hepatocellular Carcinoma

**FIGURE 8:**
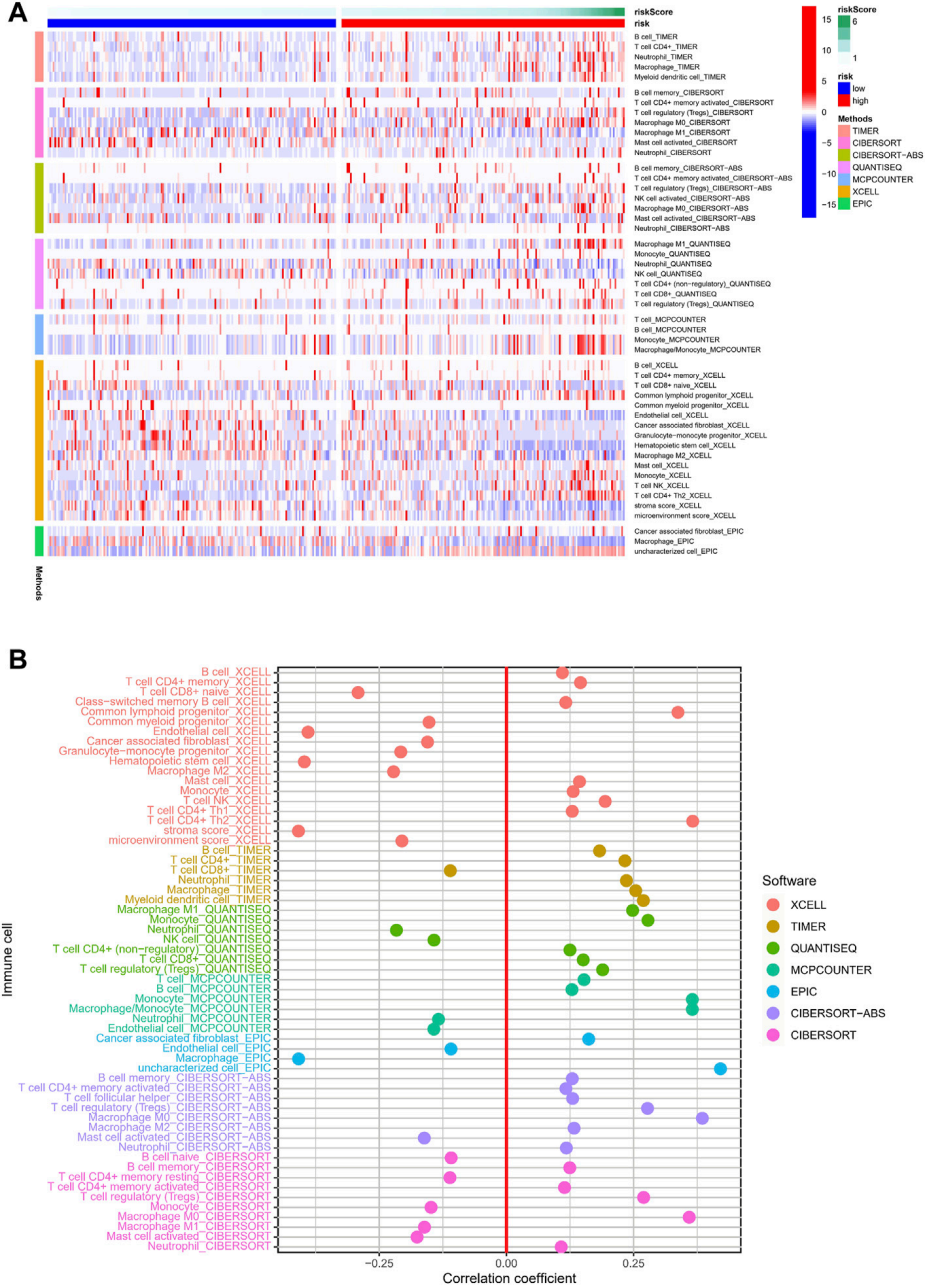
Relationship between immune cells and risk score. **(A)** TIMER, CIBERSORT, CIBERSORT-abs, QUANTISEQ, MCP-counter, XCELL, and EPIC algorithms were used to draw heat maps of immune cell infiltration of patients with different risk scores. **(B)** Correlation coefficient between immune cells and risk score.

Necroptosis is closely related to the immune signaling of tumor cells. Targeting the necroptotic process has been reported to induce the immune system to kill tumors. RIPK3, which is involved in necroptosis, can drive cells to produce inflammatory chemokines and cytokines during cell death, thereby activating killer T cells (Snyder et al., 2019). To understand the immune cell infiltration of the patients grouped by the predictive model, we used seven algorithms to draw a heat map of immune cell infiltration and found that the high-risk group had a higher immune cell infiltration status (Figure 8A). The bubble plot depicting the association of immune cell infiltration with the risk score showed increased immune cell infiltration, including CD4^+^ memory T cells, mast cells, and B cells at XCELL, CD4^+^ T cells at TIMER, T cell regulatory at QUANTISEQ, monocytes at MCPCOUNTER, and macrophages M0 at CIBERSORT, in the high-risk group (Figure 8B).

**FIGURE 9:**
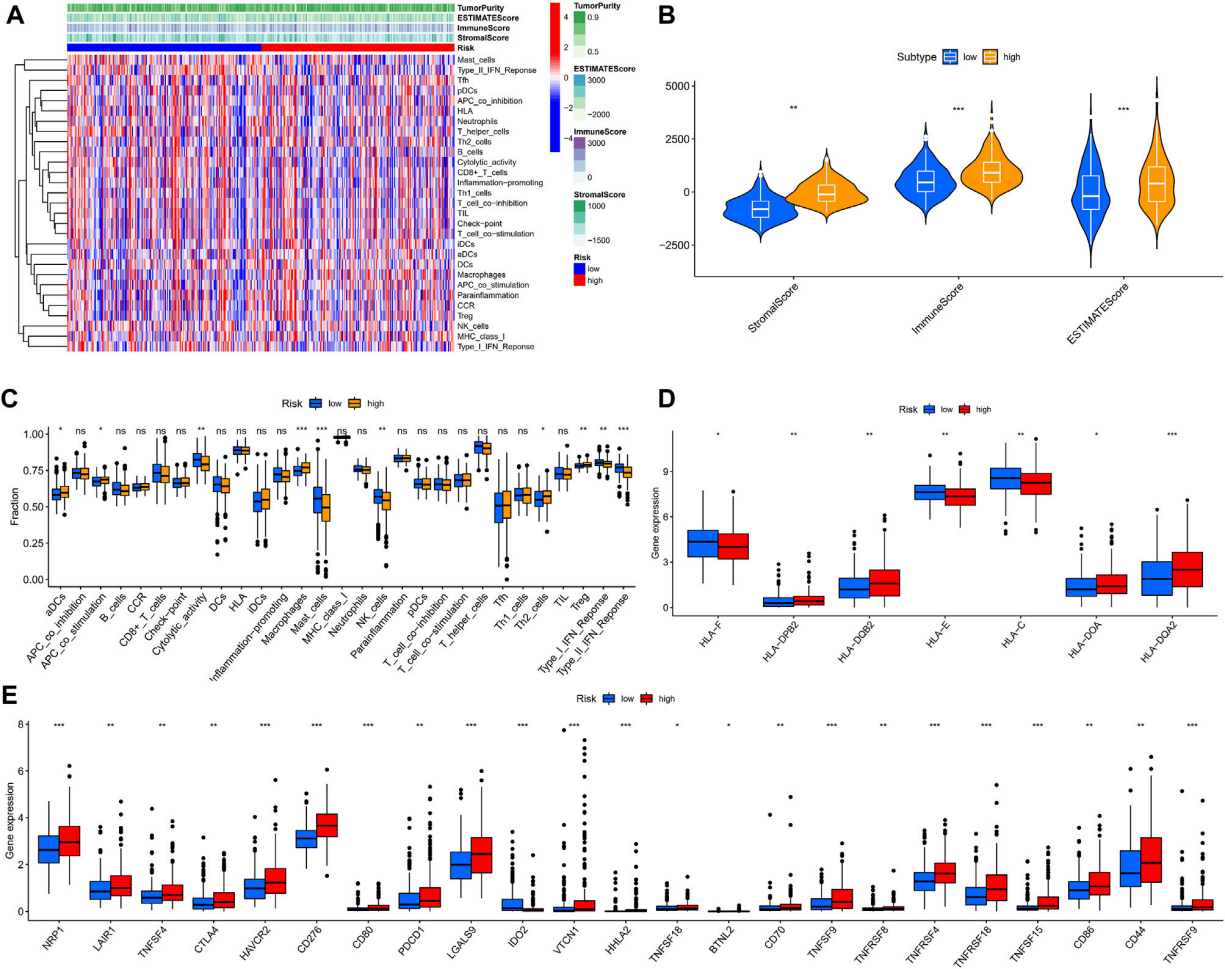
Immune microenvironment analysis in different risk groups. **(A,B)** Stromal, immune, and estimate scores of patients with different risks. **(C)** The ssGSEA scores of immune cells and immune functions. **(D)** Expression of HLAs in different risk groups. **(E)** The comparison of immune checkpoints between high and low-risk groups. (ns, *p* > 0.05; *, *p* < 0.05; **, *p* < 0.01; ***, *p* < 0.001).

We used ssGSEA to quantify the enrichment scores of the immune cell subsets and their associated functions for each sample in the TCGA cohort. The results showed apparent differences in immune cell infiltration among the different risk groups (Figure 9A). Antigen-presenting cells such as macrophages were more highly expressed in the high-risk group (Figure 9C). ESTIMATE is a tool that uses gene expression data to predict tumor purity and the presence of infiltrating stromal/immune cells in tumor tissue (Yoshihara et al., 2013). We used the ESTIMATE algorithm to evaluate the composition of immune cells in each sample by stromal score, immune score, estimated score, and tumor purity; the results showed that the high-risk group had higher stromal, immune, and estimated scores (Figure 9B). We then analyzed the expression of HLA. HLA-C, which belongs to HLA-I and can present endogenous tumor antigens to kill tumor cells effectively, was less expressed in the high-risk group, while HLA-II, such as HLA-DPB2, HLA-DQB2, HLA-DOA, and HLA-DQA2, showed an increase in the high-risk group (Figure 9D). HLA-II is mainly expressed on the surface of antigen-presenting cells, and we speculated that the increased expression of HLA-II in high-risk patients might be associated with increased immune cell infiltration in a necroptotic environment.

The analysis of HLA reflected the possible differences in the immune status and susceptibility of patients with different risk groups to immune checkpoint blockade (ICB). Given that heterozygosity of HLA-I can reflect the effectiveness of tumor ICB (Chowell et al., 2018), we conducted a differential analysis of immune checkpoints. We found that multiple immune checkpoint proteins, including programmed cell death protein 1 (PDCD1) and CTLA4, were highly expressed in the high-risk group (Figure 9E). These findings suggest that patients in the high-risk group may benefit more from ICB therapy.

### Functional Enrichment Analysis of Different Risk Groups

**FIGURE 10:**
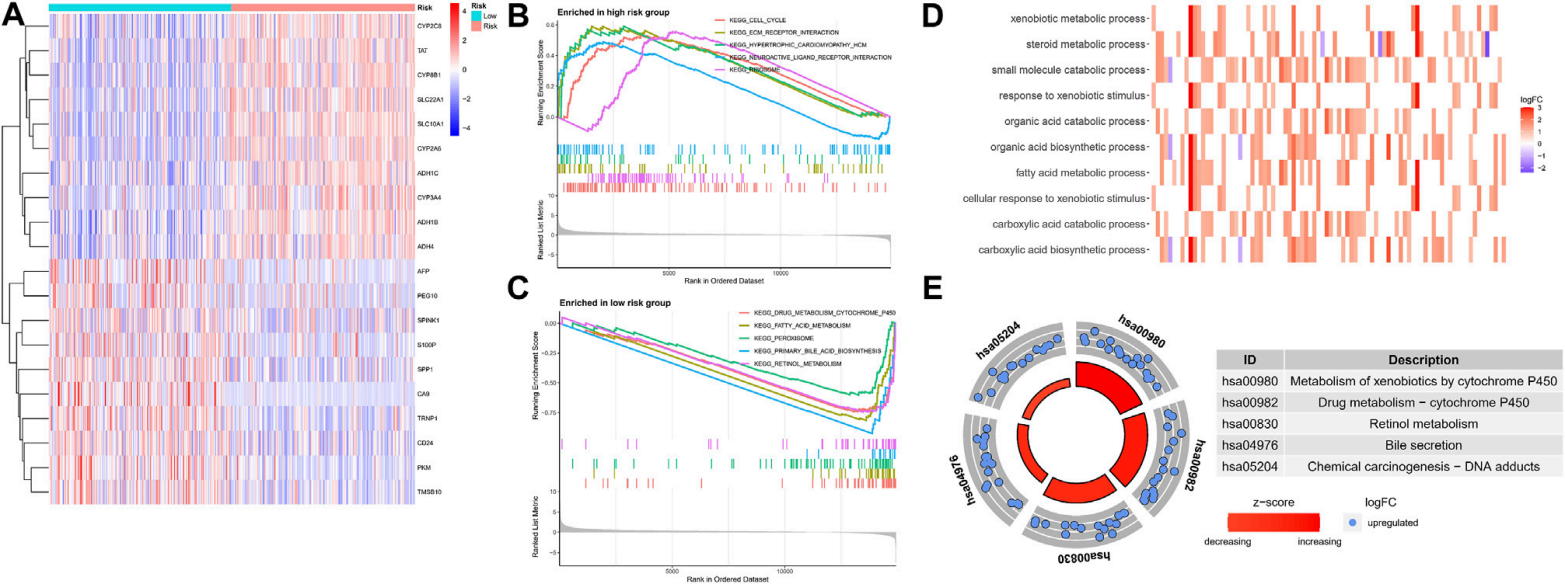
Functional enrichment analysis in different risk groups. **(A)** The heatmap of differentially expressed genes. **(B,C)** GSEA of the top 5 pathways significantly enriched in both high **(B)** and low-risk groups **(C)**. **(D,E)** GO and KEGG enrichment analysis of differentially expressed genes.

To explore the underlying molecular mechanisms of the different risk groups, we performed a differential gene expression analysis of patients in the TCGA cohort and identified 206 genes (*p* < 0.05, |log2FC| > 1) (Supplementary File S3). The top 20 differentially expressed genes are shown in a heat map in Figure 10A. The GSEA algorithm was used to detect the main enrichment pathways. Cell cycle and ECM receptor interaction were dominant in the high-risk group (Figure 10B), whereas drug metabolism, cytochrome p450, fatty acid metabolism, and peroxisome were critical pathways in the low-risk group (all *p* < 0.05, FDR <0.25, |NES| > 1.5) (Figure 10C). Immune- and metabolism-related processes accounted for most of the top 10 results of the GO enrichment analysis, such as xenobiotic metabolic process, fatty acid metabolic process, and steroid metabolic process (Figure 10D). As shown in the circle diagram of the KEGG analysis results, the top five enriched pathways mainly involved cell metabolism, drug metabolism, and drug sensitivity, including metabolism of xenobiotics by cytochrome P450, drug metabolism–cytochrome P450, retinol metabolism, bile secretion, and chemical carcinogenesis–DNA adducts (Figure 10E).

### Drug Effectiveness Analysis

**FIGURE 11:**
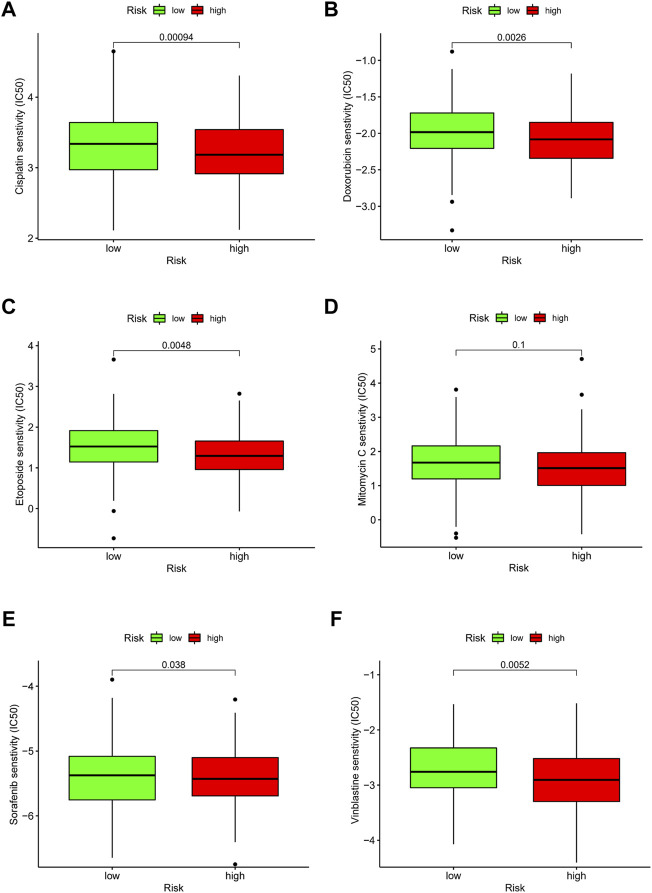
Drug effectiveness of different risk groups. **(A)** Cisplatin. **(B)** Doxorubicin. **(C)** Etoposide. **(D)** Mitomycin C. **(E)** Sorafenib. **(F)** Vinblastine. Five of the six drugs showed IC_50_ differences (*p* < 0.05).

Due to the limitations of systemic chemotherapy, most patients with advanced HCC can choose local therapy based on TACE, which delivers chemotherapy drugs to the vicinity of the tumor (Raoul et al., 2019). The enrichment analysis presented above showed that patients in the different risk groups may differ in drug metabolism and sensitivity. We quantified the IC_50_ values of six drugs commonly used for HCC and found that cisplatin, doxorubicin, etoposide, sorafenib, and vinblastine had lower IC_50_ in the high-risk group (all *p* < 0.05) (Figure 11). Cisplatin, doxorubicin, and sorafenib are the first-line drugs recommended for treating HCC in China’s standard for diagnosis and treatment of primary liver cancer (2022 edition) (National Health Commission of the people’s Republic of China, 2022). The predictive model identified in this study could be a potential predictor of chemosensitivity.

### Construction of Molecular Subtypes

**FIGURE 12:**
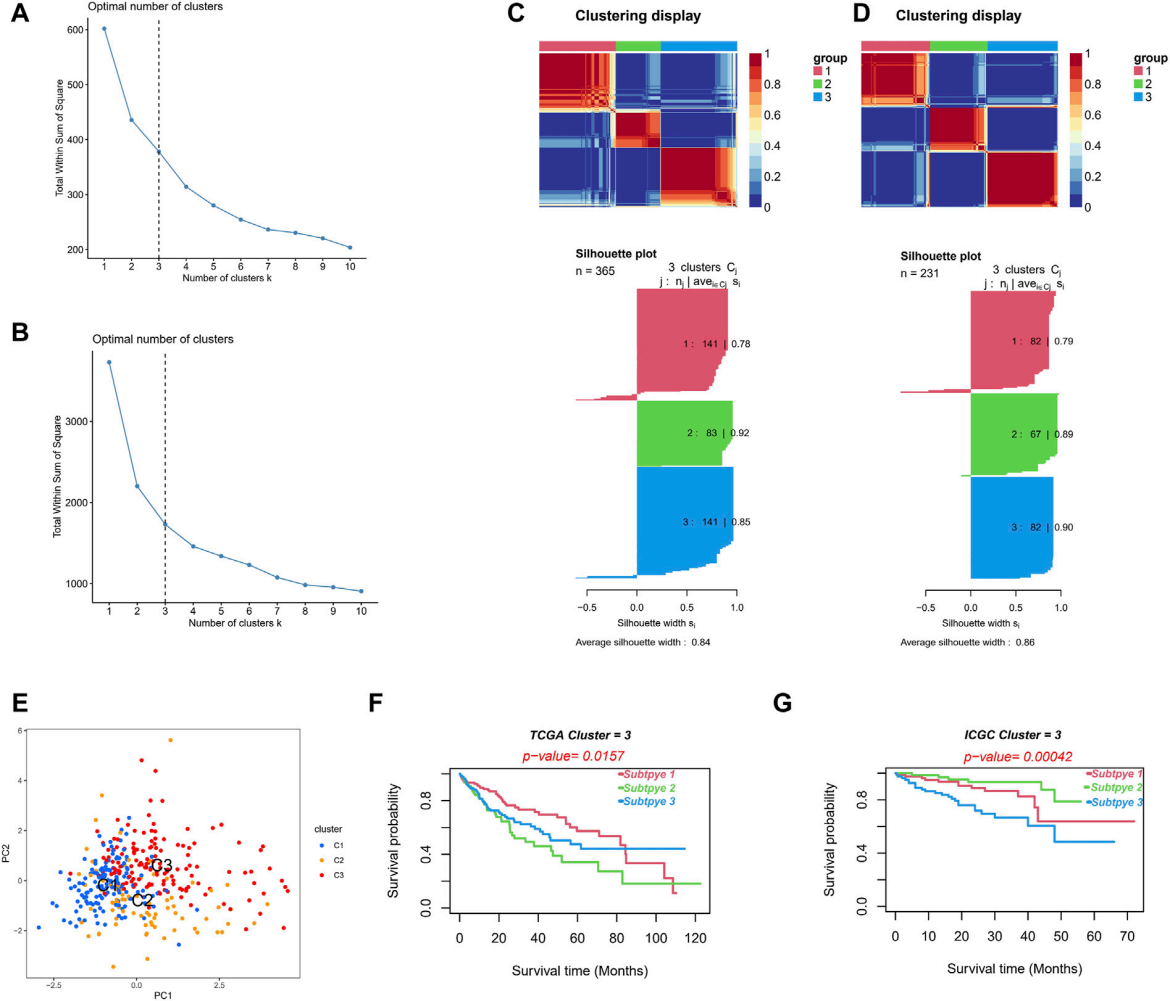
Different molecular subtypes identified by risk signature. **(A,B)** Establish the optimal cluster number k value in the TCGA **(A)** and ICGC **(B)** cohorts. **(C,D)** Patients in the TCGA **(C)** and ICGC **(D)** cohorts were divided into three clusters using the NMF clustering algorithm. **(E)** The PCA of clusters. **(F,G)** Kaplan–Meier survival curves of OS in three clusters.

We divided the TCGA cohort patients into different subtypes based on the NMF algorithm to further explore the role of NRLs in HCC progression. The optimal number of clusters k was established by calculating the cluster correlation coefficient, with k = 3 being the optimal number of clusters (Figure 12A). A consistent NMF was performed again to define three clusters, C1 (*n* = 141), C2 (*n* = 83), and C3 (*n* = 141), with an average silhouette width of 0.84 (Figure 12C).

Consistent NMF was also performed on the validation set (ICGC cohort), and k = 3 was the optimal number of clusters (Figure 12B). We identified three clusters, C1 (*n* = 82), C2 (*n* = 67), and C3 (*n* = 82), with an average silhouette width of 0.86 (Figure 12D). The PCA analysis showed obvious distinctions in the different two-dimensional distribution maps of the three clusters (Figure 12E). The subtype matching model of the TCGA and ICGC cohorts was identified through the subclass algorithm; we determined the following: TCGA-C1 = ICGC-C2; TCGA-C2 = ICGC-C3; and TCGA-C3 = ICGC-C1. Moreover, the NTP algorithm suggested that the high-risk subgroup in the TCGA cohort (k = 0.774, *p* < 0.001) and in the ICGC cohort (k = 0.679, *p* < 0.001) could better correspond to the cluster C2, with the OS of the C2 being significantly lower than that of C1 and C3 in both cohorts (log-rank test *p* < 0.05, Figures 12F,G). The clusters constructed based on the NMF algorithm and prognosis-related NRLs showed better prediction skills concerning survival. We believe that different molecular subtypes can provide another insight into distinguishing patients.

### Validation of the Necroptosis-Related Long Noncoding RNAs in Hepatocellular Carcinoma Tissues and Cell Lines

**FIGURE 13:**
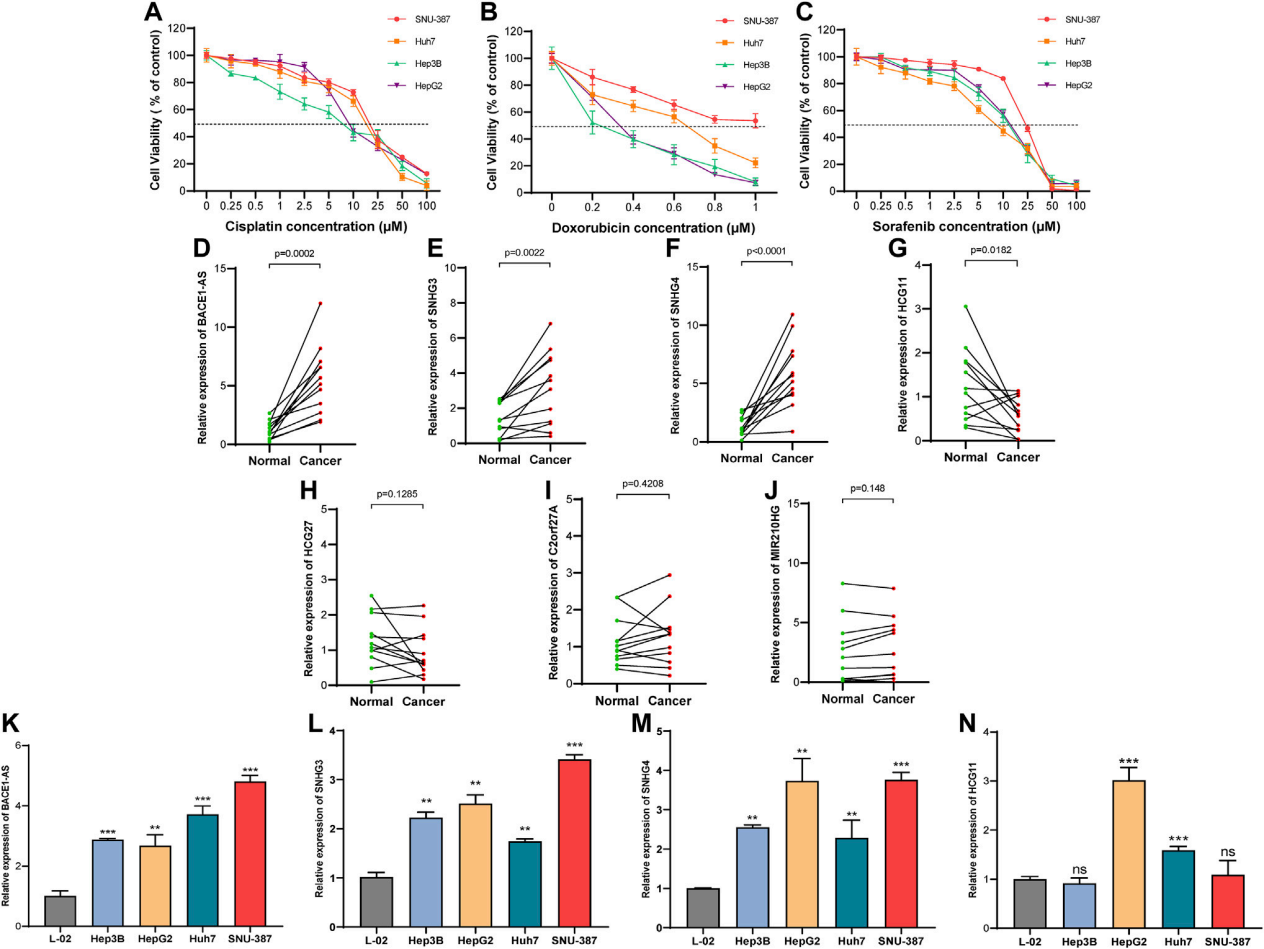
Expression of NRLs in HCC patients and different drug-sensitive cell lines. **(A–C)** Cell viability in the four HCC cell lines after cisplatin, doxorubicin, and sorafenib treatment for 48 h. Data are presented as the mean ± standard deviation (*n* = 5). **(D–J)** Relative expression of seven NRLs in HCC patients. **(K–N)** Expression of BACE1-AS, SNHG3, SNHG4, and HCG11 in cell lines. (ns, *p* > 0.05; *p < 0.05; **p < 0.01; ***p < 0.001; student t-test).

We collected 12 pairs of HCC tissues and paracancerous tissues from the Affiliated Tumor Hospital of Nantong University to verify the expression of the seven NRLs in the signature. We performed RT-PCR after the extraction of total RNA from tissues and found that three out of five high-risk (HR > 1, Figure 3B) NRLs (BACE1-AS, SNHG3, SNHG4) were more expressed in HCC tissues than in paracancerous tissues (all *p* < 0.01, Figures 13D–F). As a protective factor, the expression of HCG11 in HCC was lower than that in adjacent tissues (*p* < 0.05, Figure 13G), which was consistent with the results from the TCGA database. The three remaining lncRNAs (HCG27, C2orf27A, MIR210HG) were not significantly different (Figures 13H–J).

Next, we examined the expression of these four NRLs (BACE1-AS, SNHG3, SNHG4, HCG11) in different drug-sensitive HCC cell lines and liver cell lines. Among the five drugs analyzed above (Figure 11), cisplatin, doxorubicin, and sorafenib are commonly used for treating HCC. We selected SNU-387, Huh7, Hep3B, and HepG2 cell lines to detect the cell viability at different drug concentrations (Figures 13A–C). The IC_50_ values are shown in Table 2. The IC_50_ values of the three drugs of SNU-387 were the highest among the four cell lines, indicating that this cell line had apparent resistance to the commonly used drugs. RT-PCR results showed that the expression levels of SNHG3, SNHG4, and BACE1-AS in SNU-387 were higher than those in the other four cell lines (all *p* < 0.001), and the expression in normal hepatocytes was the lowest (Figure 13K–M). The expression of HCG11 as a protective factor was the highest in HepG2 cells, but the expression level in other HCC cell lines, including SNU-387, was not significantly different from that in normal hepatocytes (Figure 13N).

**TABLE 2:** IC_50_ values for cisplatin, doxorubicin, and sorafenib treatment in HCC cell lines.

Cell line	IC_50_ values of drugs (μM)^a^		
	Cisplatin	Doxorubicin	Sorafenib
SNU-387	17.047 (15.25–18.844)	1.055 (0.941–1.17)	21.496 (19.884–23.108)
Huh7	13.409 (11.857–14.962)	0.547 (0.488–0.606)	7.571 (6.577–8.566)
Hep3B	6.7 (5.631–7.768)	0.241 (0.197–0.284)	10.878 (9.846–11.909)
HepG2	12.174 (10.89–13.457)	0.326 (0.309–0.343)	11.971 (10.959–12.984)

a IC_50_ values indicate the cisplatin, doxorubicin, and sorafenib concentration [μM; mean (95% CI)].

## Discussion

Despite the variety of treatment options, OS in patients with HCC remains poor. It is important to understand what hinders the progress of HCC treatment. TME may be an essential factor in the occurrence and development of HCC (Krishnan et al., 1985). Some traditional grading systems, such as TNM grading and Barcelona staging, neither reflect the TME of HCC patients nor accurately predict the patient’s prognosis. As an important part of TME, the infiltration by immune cells also affects the benefits of HCC immunotherapy to a certain extent (Yu et al., 2020), with many clinical trials of immunotherapy related to HCC having been conducted globally (Sangro et al., 2013; Kudo et al., 2021). However, the TME may cause an inadequate response and limited therapeutic efficacy when using immunotherapy (El-Khoueiry et al., 2017; Kudo et al., 2021). It is therefore necessary to continue exploring the role of immune factors in the treatment of HCC. It has been reported that necroptosis may alter the TME, thereby affecting the type of liver cancer (Saeed and Jun, 2019). However, its specific role and impact on the prognosis of HCC patients remain unclear. In this study, a prognostic model was constructed based on necroptosis-related lncRNAs, and the patients were grouped into high- and low-risk groups. We systematically investigated differences in immune cell infiltration, immune checkpoints, HLA, and drug sensitivity among the different subgroups and constructed HCC molecular subtypes based on the NMF algorithm. Finally, we used RT-PCR to verify the expression levels of NRLs in tissues and cells.

We analyzed the expression of 67 NRGs in HCC, of which 12 of 19 screened were found to be upregulated and seven were downregulated. Nineteen NRGs and all annotated lncRNAs were subsequently analyzed, and 508 NRLs were identified. We constructed the prognostic signature containing seven NRLs and validated it using an external cohort. The biological function of these NRLs is associated with the progression of HCC. BACE1-AS can promote abnormal proliferation, cell cycle progression, migration, invasion, and apoptosis of HCC through the miR-214-3p/APLN axis (Tian et al., 2021). In HCC cell lines, SNHG3 overexpression promotes the proliferation, migration, and EMT, and inhibits apoptosis (Zhao et al., 2019), while higher levels of SNHG4 are more likely to indicate poor prognosis in liver cancer (Jiao et al., 2020). Through transcriptomic analysis, some studies have suggested that C2orf27A can affect the resistance of HCC cells to sorafenib through immune infiltration (Yuan et al., 2021), which is consistent with our findings. MIR210HG can be used as a glycolysis-related lncRNA to influence the progression of HCC (Xia et al., 2021). We plotted nomograms to predict 1-, 3-, and 5-year OS in HCC patients to intuitively use this predictive model. It was clear from the ROC curves that the predictive model built with NRLs was accurate and reliable.

After measuring the single-cell transcriptomic profiles of HCC biological samples from 19 patients, Wang’s team found that the heterogeneity of HCC TME significantly affected the treatment response and prognosis (Ma et al., 2019). Therefore, it is necessary to deeply understand the role of TME in HCC. It has been shown that necroptosis is involved in CD4^+^ T cell–mediated endothelial cell death (Kwok et al., 2017). Our results showed that the risk score positively correlated with the CD4^+^ T cell infiltration level, given that the high-risk group had more Th2 and Tregs. We speculate that necroptosis may promote increased CD4^+^ T cells in the TME. Moreover, the risk score positively correlated with macrophage levels, and the high-risk group had more macrophages, which may have been caused by increased activation of RIPK3 in the inflammatory environment formed by necrotizing apoptosis (Hao et al., 2021). There were fewer NK cells and mast cells in the high-risk group, and the mechanism is unknown, which needs further exploration. Immune cells and stromal cells are two major non-tumor components of TME that can modulate the sensitivity of immunotherapy by affecting tumor purity. Low purity may be linked to increased immune evasion and poor prognosis (Gong et al., 2020). We found that the high-risk group had higher immune, stromal, and lower tumor purity, which indicates that patients in the high-risk group may benefit more from immunotherapy. HLA-I is plays an essential role in the cytotoxic T-lymphocyte–mediated response, presenting antigens to CD8^+^ T cells (Durgeau et al., 2018). The ability of HLA-I class molecules to present antigens is related to the degree of heterozygosity of HLA alleles (Chowell et al., 2019). We found an interesting phenomenon where HLA-DPB2, HLA-DQB2, HLA-DOA, and HLA-DQA2, which belong to HLA-II, showed increased expression in the high-risk group, while HLA-C, which belongs to HLA-I, showed decreased expression. In this study, we only found that HLA-C expression was decreased in high-risk patients; we cannot speculate whether high-risk patients have a decrease in ICB treatment sensitivity, and this topic needs further research. However, most of the immune checkpoint proteins, including PDCD1 and CTLA4, were highly expressed in the high-risk group, which may suggest that the high-risk group may have better ICB treatment effects.

GSEA analysis showed that the high-risk group was mainly enriched in pathways such as cell cycle and ECM receptor interaction. Necroptosis is a specialized form of cell death, which interacts with the cell cycle *via* interferons (Frank et al., 2019). The ECM–receptor interaction pathway regulates the processes of tumor shedding, adhesion, movement, and hyperplasia (Bao et al., 2019). Through specific key mediators (Gong et al., 2019), necroptosis has been identified to promote tumor metastasis and progression (Gong et al., 2019). These findings demonstrate the credibility of GSEA analysis. Significant enrichment in cellular metabolic pathways, including fatty acid metabolism, steroid metabolism, and drug metabolism, was found in the GO and KEGG analyses. It has been reported that ceramides and very long-chain fatty acids accumulate during necroptosis (Parisi et al., 2017), which is consistent with our pathway analysis results. Cisplatin belongs to platinum, which can covalently bind with DNA, inhibit DNA replication, and promote cell cycle arrest. Both cisplatin and doxorubicin upregulate RIPK3, which binds and phosphorylates calmodulin kinase II (CaMKII), thereby regulating the opening of the mitochondrial permeability transition pore (mPTP) and leading to necroptosis (Christidi and Brunham, 2021; Sazonova et al., 2021). Combined with our results, patients in the high-risk group may be more sensitive to cisplatin and doxorubicin. Sorafenib is a protein kinase inhibitor with activity inhibition of many protein kinases, including vascular endothelial growth factor receptor (VEGFR), platelet-derived growth factor receptor (PDGFR), and Raf protein kinase. Heat shock protein 90α (HSP90α) promotes sorafenib resistance in HCC by inhibiting necroptosis under hypoxia (Liao et al., 2021). Finally, we detected NRLs in 12 pairs of tissues. Contrary to our results and TCGA analysis, some studies have reported higher expression of HCG11 in HCC than in adjacent tissues (Xu et al., 2017; Li et al., 2019), which may be caused by differences between different regions and ethnic groups. The expression levels of three high-risk lncRNAs, BACE1-AS, SNHG3, and SNHG4, were significantly higher in SNU-387 than in other cell lines, including normal hepatocytes. It was confirmed from the cellular and tissue levels that the lncRNAs included in the signature may be related to the occurrence, development, and drug resistance of HCC.

This study analyzed the model’s predictive performance from various perspectives, including immunotherapy sensitivity, chemotherapy drug sensitivity, and OS. lncRNA was for the first time combined with necroptosis in HCC, with consensus clustering being used for HCC with the assistance of the NMF algorithm. Compared with other necroptosis-related prediction models (Wang and Liu, 2021; Zhao et al., 2021), we used ICGC data for external validation. HCC cells with different drug sensitivity were used for *in vitro* validation. We compared the C-index values of several latest prediction signatures in HCC with ours (Lin et al., 2022; Miao et al., 2022; Ye et al., 2022; Zhao et al., 2022; Zhou et al., 2022) (Supplementary Figure S2). Genes in the prediction signature are listed in Supplementary File S4. Our signature has the highest C-index of the five signatures included, indicating that it performs better in terms of prediction.

There are still several limitations in our study. The primary datasets were obtained from public databases, and more real-world data are needed to validate the clinical value of the signature. Moreover, we only compared the IC_50_ between the high-risk and low-risk groups on several commonly used drugs due to insufficient data on GDSC. This study has not yet elucidated how lncRNA regulates necroptosis in HCC, which requires further research.

## Conclusion

We constructed a necroptosis-related prognostic signature that can be used to assess the prognosis and TME status of HCC patients. Combined with preliminary validation at the tissue and cellular levels, the signature could provide an option for individualized patient treatment and prognostic assessment. The potential relationship between necroptosis and lncRNA may be a key to immunotherapy for HCC, but the mechanisms deserve further investigation.

## Data Availability

The original contributions presented in the study are included in the article/Supplementary Material, further inquiries can be directed to the corresponding authors.
