# Supplementary material for: Identification and Validation of Necroptosis-Related LncRNA Signature in Hepatocellular Carcinoma for Prognosis Estimation and Microenvironment Status
Source: Front Genet. 2022 Jun 8;13:898507. doi: 10.3389/fgene.2022.898507 (PMC9214229; doi:10.3389/fgene.2022.898507)
Supplement: Supplementary file 5 [file DataSheet1.DOCX]

Supplementary Material


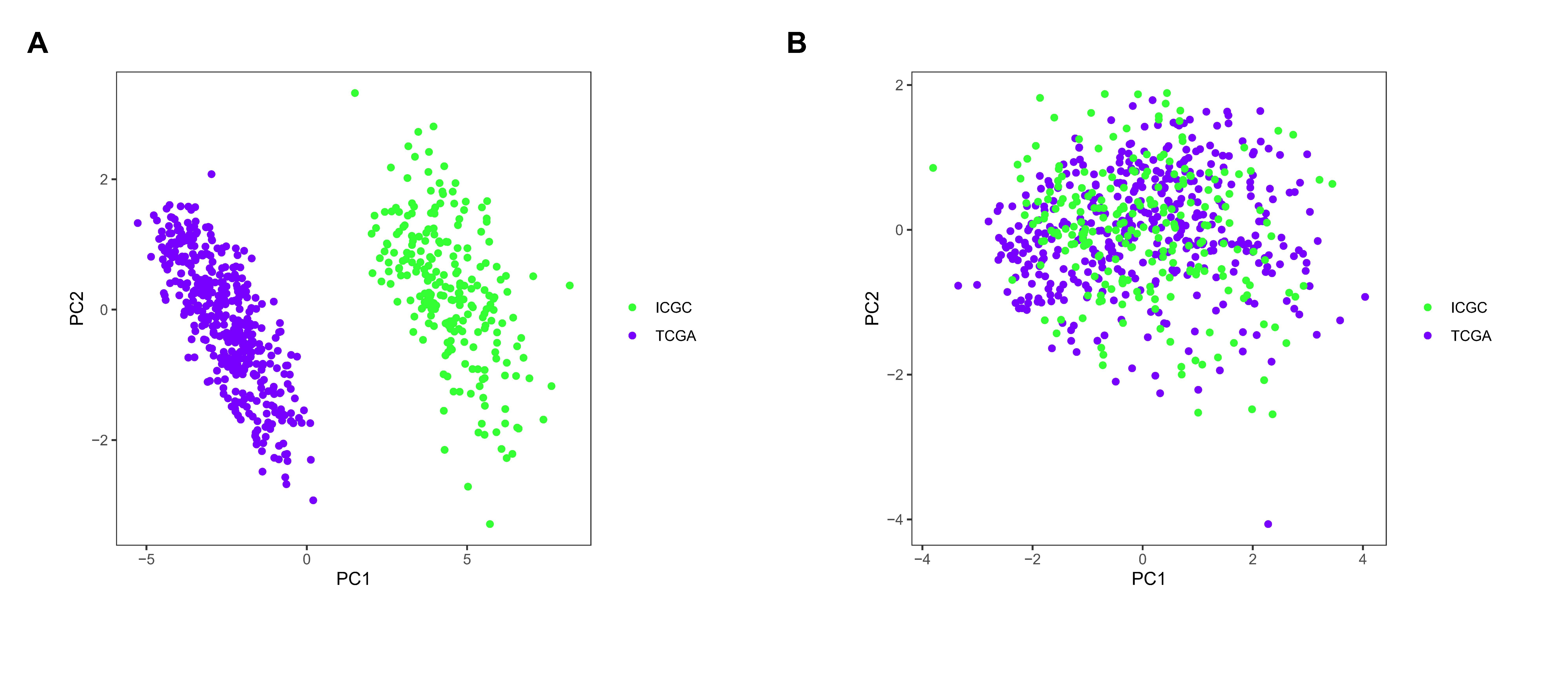


**Figure S1.** PCA plot of the TCGA and ICGC cohort. **(A)** PCA plot before correction. **(B)** PCA plot after correction.

**Figure S2.** Comparation of C-index among different signatures.
